# Enhancement of spontaneous emission in Tamm plasmon structures

**DOI:** 10.1038/s41598-017-09245-7

**Published:** 2017-08-21

**Authors:** A. R. Gubaydullin, C. Symonds, J. Bellessa, K. A. Ivanov, E. D. Kolykhalova, M. E. Sasin, A. Lemaitre, P. Senellart, G. Pozina, M. A. Kaliteevski

**Affiliations:** 1 St Petersburg Academic University, 8/3 Khlopina Str, St Petersburg, 194021 Russia; 2 Univ Lyon, Université Claude Bernard Lyon 1, CNRS, Institut Lumière Matière, F-69622 LYON, France; 3 ITMO University, 49 Kronverksky Pr., St. Petersburg, 197101 Russia; 4 Ioffe Institute, 26 Politekhnicheskaya, St. Petersburg, 194021 Russian Federation; 5 Centre de Nanosciences et Nanotechnologies, CNRS Université Paris-Saclay, Route de Nozay, F-91460 Marcoussis France; 6 Department of Physics, Chemistry and Biology, Linköping University, 58183 Linköping, Sweden

## Abstract

It was theoretically and experimentally demonstrated that in metal/semiconductor Tamm plasmon structures the probability of spontaneous emission can be increased despite losses in metal, and theoretical analysis of experimental results suggested that the enhancement could be as high as one order of magnitude. Tamm plasmon structure with quantum dots has been fabricated and the emission pattern has been measured. Electromagnetic modes of the structure have been analyzed and modification of spontaneous emission rates has been calculated showing a good agreement with experimentally observed emission pattern.

## Introduction

Absorption of light in metals is usually considered as an objection for utilization of metallic elements in optical schemes of optoelectronic devices^1^. Even in the noblest metals (silver and gold) the absorption of light in the visible – infrared range is substantial^2^ and, thus, their properties differ from those of a perfect conductor.

The main obstacle preventing the application of metallic mirrors in optoelectronics is heating of the metal due to optical absorption, which leads to a severe degradation of the mirrors and surrounding materials. Furthermore, such absorption is considered to be detrimental for optical coherence and therefore it prevents the development of even low output power lasers.

Absorption in metals significantly reduces the performance of prospective devices based on metamaterials, such as optical interconnectors and devices based on the effects of negative refraction^3–6^. At present, the most widespread practical applications of plasmonic structures are surface enhanced Raman scattering^7, 8^ and other applications for biosensoring^9–11^.

In recent years two major breakthroughs in metallic optoelectronic devices have been made. First, the room temperature generation of electrically pumped sub-wavelength metallic-cavity semiconductor lasers has been demonstrated^12^. Second, macroscopic optical coherence^13^, lasing^14^, and single photon emission^15^ have been demonstrated in Tamm plasmon based structures^16–18^. Tamm plasmon is the state of the electromagnetic field localized on the interface between metal and Bragg mirror of peculiar design. Tamm plasmon state in metal/dielectric layered structures can be designed in the way that provide positioning of the node of electric field at the metallic layer thus suppressing the absorption of light^19^.

Modification of spontaneous emission rates in the structures with inhomogeneous dielectric constant (Purcell effect) is an important property defining the efficiency of optoelectronic devices^20^. The Purcell effect can be considered as a consequence of interference in the structure^21^, and since for Tamm plasmon structures interference is weakly influenced by absorption in metal, one can expect substantial increase of probability of spontaneous emission for Tamm plasmon structures.

**Figure 1:**
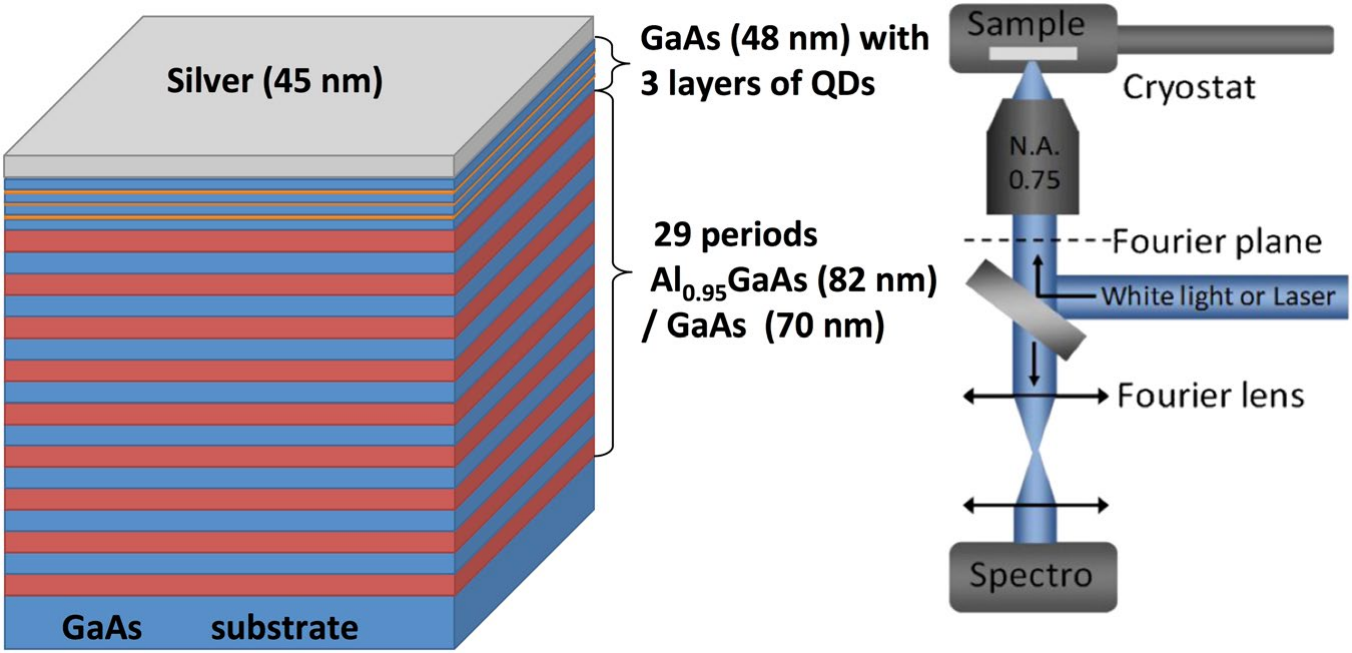
Scheme of the structure and experimental set-up.

This paper is aimed at experimental and theoretical investigation of spontaneous emission rates in Tamm plasmon based microcavities where the InAs quantum dots active area is sandwiched between GaAs/AlAs Bragg reflector and silver layer (See Fig. 1). For the details of fabrication process and precise description of the structure see section Methods.

## Results and Discussion

To analyze the modification of spontaneous emission rates we have compared the calculated modal Purcell factor *F*(*ω*, *θ*) pattern (ratio of probability of spontaneous emission for specific frequency and direction of emission to those in uniform media) and the experimentally measured photoluminescence pattern.

**Figure 2:**
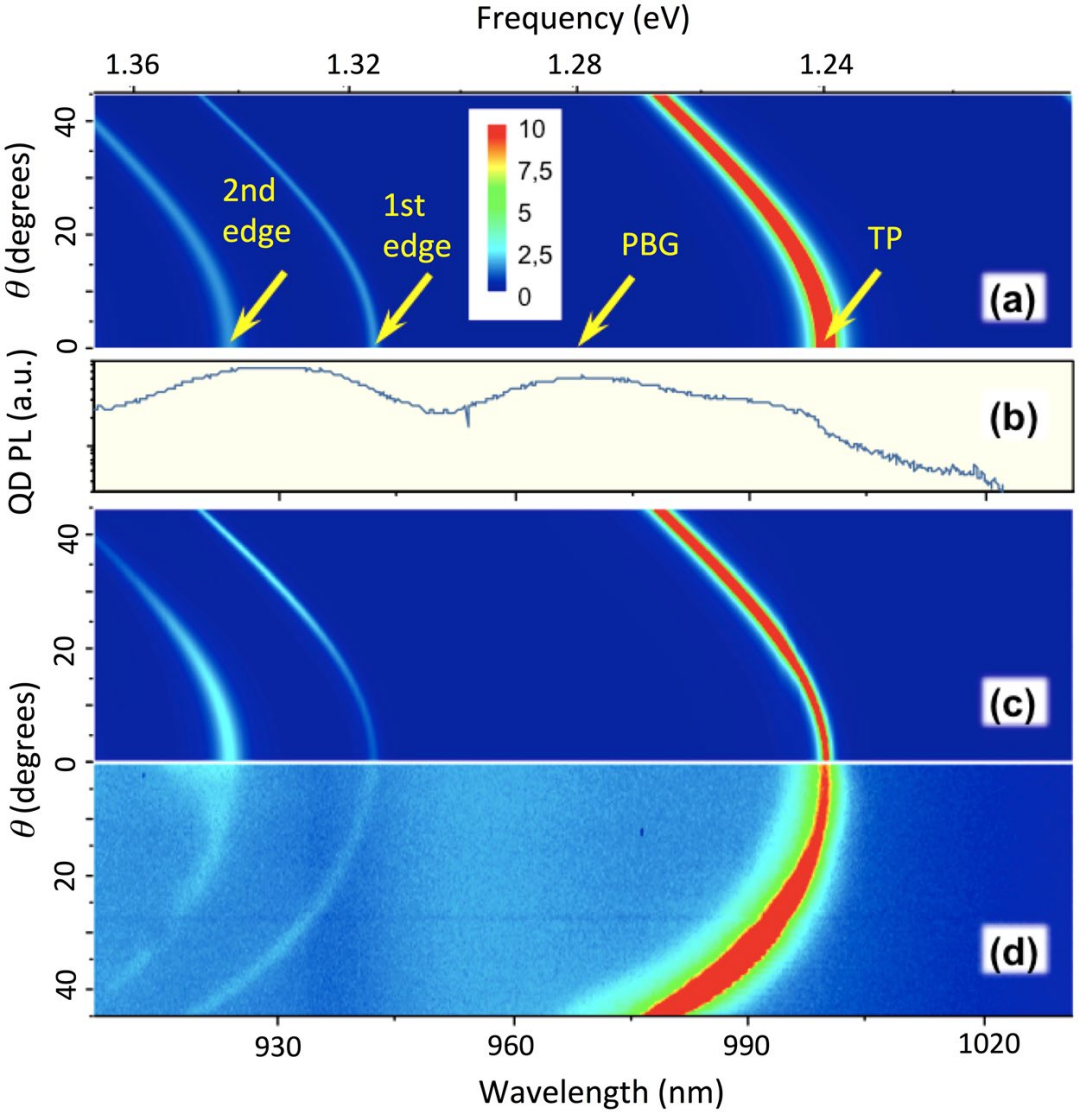
(**a**) Dependence of the calculated modal Purcell factor on frequency and angle of emission; (**b**) Emission spectrum of quantum dots; (**c**) Dependence of the product of the calculated modal Purcell factor and the emission spectra of quantum dots on frequency and angle of emission; (**d**) Experimentally measured photoemission spectra. Arrows mark the modes (TP –Tamm plasmon, PBG – photonic band gap, 1^st^ edge – 1^st^ edge state, 2^nd^ edge – second edge state).

It is essential that the layers of InAs quantum dots serve as an emitter. Quantum dots possess a wide luminescence band and a large ionization energy exceeding 100 meV, while *kT* at room temperature corresponds to 25 meV. The capture of non-equilibrium excitons and holes by quantum dots is an extremely fast process (the capture time is below 1 ps) while thermal ionization time of quantum dots is above 100 ns^22^. Radiative emission rate of quantum dot can be estimated as:

1 $$W \sim \frac{1}{5}{\alpha }^{2}{a}_{B}\frac{4n{\omega }^{3}}{{c}^{2}}$$

where *α*_*B*_ is the size of the area where electron and holes are localized^23^, and for the considered quantum dots corresponds to the radiative lifetime of ~1 ns. In other words, charge carriers captured by quantum dot emitted at a specific energy, cannot escape QD and need to be re-captured by another quantum dot emitting a light photon of different energy. Therefore, the emission pattern of Tamm plasmon structures (see Fig. 2) with quantum dots will be defined by the modal Purcell factor and luminescence spectrum

2 $$I(\omega ,\theta )=F(\omega ,\theta )\rho (\omega )$$

where *ρ*(*ω*) is the emission spectrum of bare quantum dots, see section Methods.

Figure 2a shows the modal Purcell factor as a function of emission direction and frequency for the quantum dots layers shown in Fig. 1a, calculated by S-quantization formalism^24^. Such formalism (see section Method) provides a self-consistent and straightforward way for calculating the probability of the spontaneous emission rate for a specific frequency, direction of emission and polarization of light, and allows to avoid the problems related to divergence of the integral describing effective mode volume^25^. It can be seen, that spontaneous emission is suppressed in all directions of propagation and frequencies except for three modes demonstrating parabolic dispersion: the low energy mode (Tamm plasmon modes) and two higher energy modes corresponding to edge state of the Bragg reflector^26, 27^. It can be seen, that for the edge states the emission rate is similar to the value for uniform media, while for the Tamm plasmon mode, the probability of spontaneous emission is increased by a factor of about 20 with respect to the free uniform space.

It is interesting to compare theoretical estimates provided by S-quantization formalism with experimental results. Figure 2b shows the emission spectrum taken from the edge of the same sample. Such spectrum is a superposition of emissions of quantum dots positioned at different distances from the edge boundary of the sample, and for each distance, the spontaneous emission pattern will differ. However, averaging of the emission pattern over the distance from the boundary provides a uniform emission pattern (see subsection *Determination of the emission spectrum of bare quantum dots* in the *Methods for details*). Thus, the emission spectra taken from the edge of the sample closely corresponds to emission spectra of bare quantum dots. PL spectrum in Fig. 2b is similar to the typical emission spectrum of bare quantum dots^22, 28, 29^ with total width exceeding 150 meV, which is composed by two wide bands (corresponding to quantum dots grounds state and excited states) centered near 1.28 eV and 1.34 eV. Such a wide spectrum provides the ideal opportunity for the direct determination of the modal Purcell factor using eq. (2).

It can be seen, that the product of the modal Purcell factor (Fig. 2a) and the emission spectrum of quantum dots (Fig. 2b) shown in Fig. 2c, closely match the experimentally observed emission pattern shown in Fig. 2d, which confirms the validity of the estimates provided by S-quantization model. Note that there are differences between calculated modal Purcell factor (Fig. 2a) and measurement emission pattern. For example, for the Tamm plasmon mode, the maximal value of modal Purcell factor is achieved for the emission angle of 0 degrees, while for the experimental emission maximum occurs for the emission angle of 50 degrees. At the same time, the maximum of the product *F*(*ω*,0)*ρ*(*ω*) corresponds to the maximum in experimental emission pattern.

The quantitative correspondence between the experimental emission and the theoretical estimate of *F*(*ω*,0)*ρ*(*ω*) is illustrated in the Table 1, providing experimentally observed values of the emission intensities for Tamm plasmon modes, 1st and 2nd edge state for the emission angle zero degrees, and values of the product of emission spectrum (Fig. 2b) and modal Purcell factor (Fig. 2a). All the values are normalized to the value corresponding to Tamm mode.

**Table 1:** Intensity of emission at normal direction and product *F*(*ω*,0)*ρ*(*ω*) normalized to the values corresponding to Tamm plasmon.

Frequency (eV)	*F*(*ω*,0)*ρ*(*ω*)	Experimental emission intensity (a.u)	Comment
1.242	1	1	Tamm plasmon
1.328	0.085	0.15	1st edge state
1.355	0.15	0.23	2^nd^ edge state
1.310	0.0004	0.05	PBG

It can be seen, that experimentally observed and theoretically estimated values correspond with the accuracy about 20%.

Within PBG, the experimentally measured signal exceeds the theoretical estimate substantially, which shows the noise level in experimental set-up. The relative intensities of emission for different angles and frequencies *I*(*ω*,*θ*) closely repeat calculated modal Purcell factor *F*(*ω*,*θ*) which provide another confirmation for S-quantization method.

**Figure 3:**
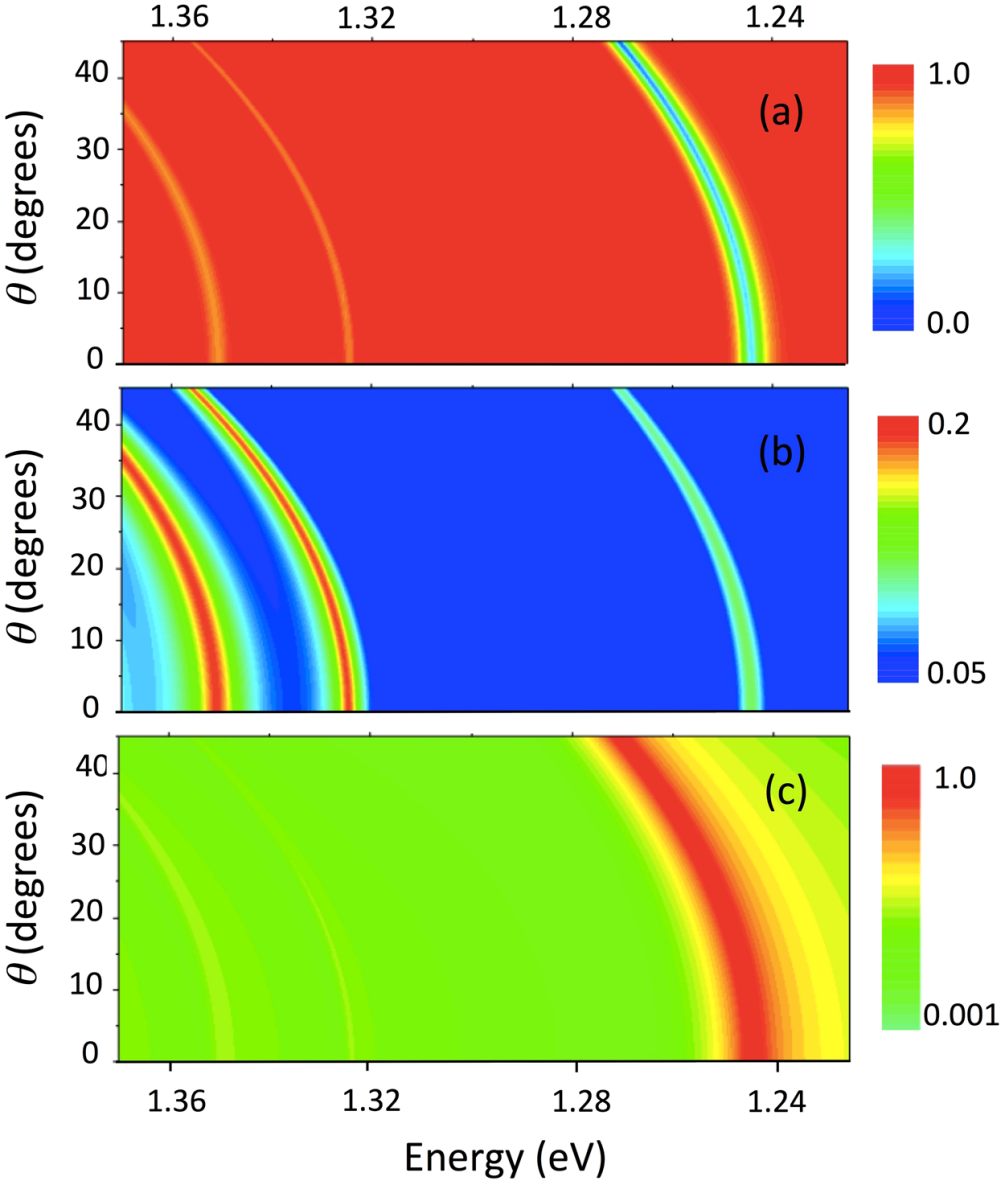
Calculated dependence of reflection (**a**), transmission (**b**) and absorption (**c**) spectra on angle and frequency of emission. Dependences on (**b**) and (**c**) are shown in logarithmic scale.

Figure 3 shows the calculated angular and spectral dependence of reflection, transmission and absorption coefficients of the structure. It can be seen that these patterns closely mimic the emission pattern. There are three branches for each pattern but they are pronounced in different manner. Tamm plasmon mode is most pronounced in the reflection and absorption spectra, while the edge states are visible in transmission.

**Figure 4:**
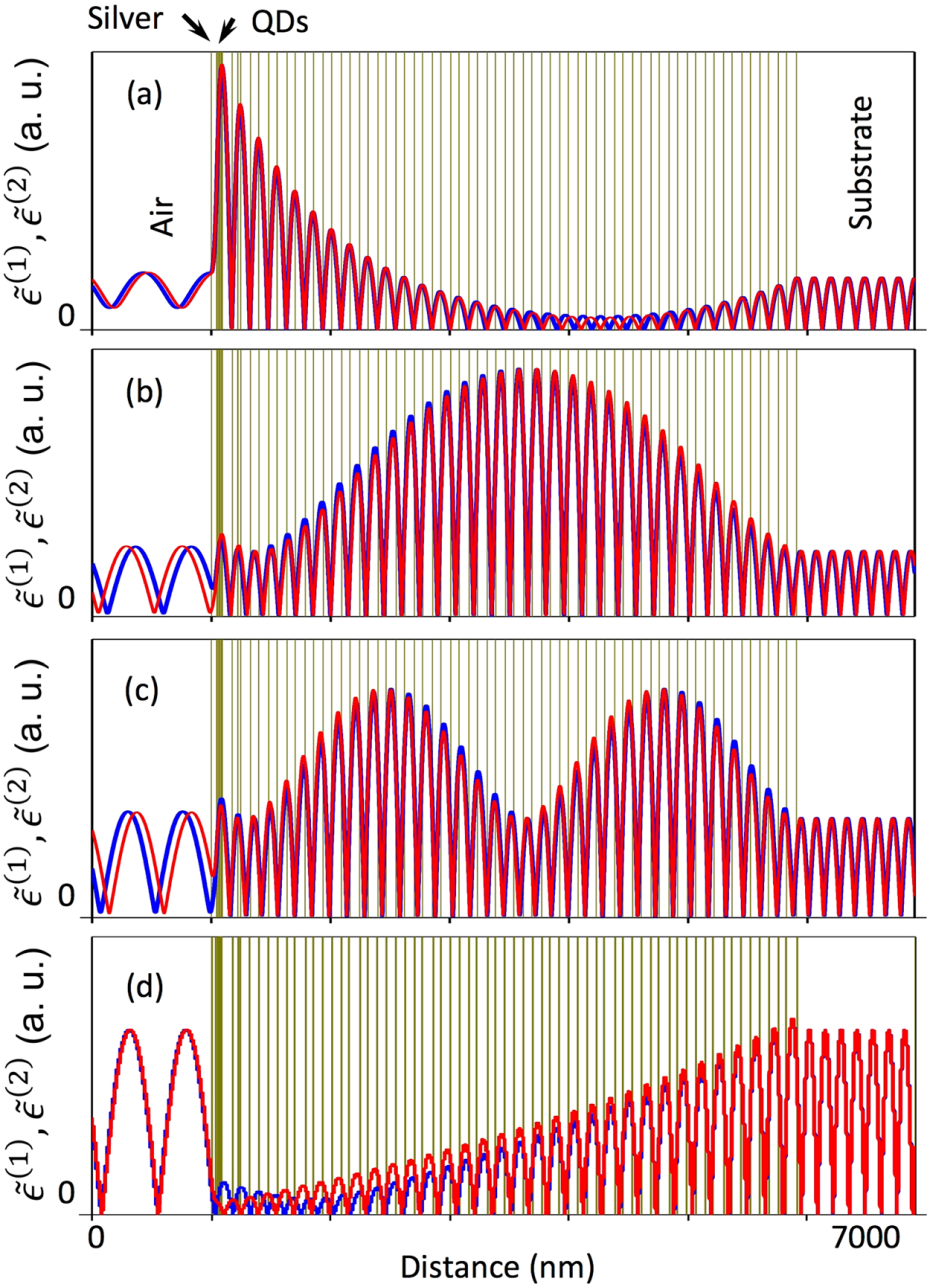
Functions $|{\tilde{{\epsilon }}}^{(1)}|$ (blue lines) and $|{\tilde{{\epsilon }}}^{(2)}|$ (red lines) for: (**a**) Tamm plasmon mode at normal incidence (*θ* = 0, $\hslash \omega \,=$ 1.2462 eV); (**b**) edge state (*θ* = 0, $\hslash \omega \,=\,$1.3247 eV); (**c**) edge state (*θ* = 0, $\hslash \omega \,=$ 1.3509 eV); (**d**) photonic band gap (*θ* = 0, $\hslash \omega \,=\,$1.31 eV). Corresponding points at emission pattern are shown by arrows at Fig. 2a.

The nature of the modification of the spontaneous emission rate in Tamm plasmons structures can be illustrated by spatial profiles of electric fields corresponding to different modes in Fig. 4. In the framework of the S-quantization formalism, there are two eigen-modes for each frequency, ${\tilde{{\epsilon }}}^{(1)}$ and ${\tilde{{\epsilon }}}^{(2)}$ “symmetric” and “antisymmetric” (see section Methods), and modal Purcell factor in a specific point is defined by modification of the sum of modula squared of these two functions. The modes of the electromagnetic field, corresponding to these functions are considered as elementary quantum oscillators with energy $\hslash \omega /2$. When the quantization box is large enough, major contribution to normalization integral is provided by the branches of the functions ${\tilde{{\epsilon }}}^{(1)}$ and ${\tilde{{\epsilon }}}^{(2)}$ in empty parts of quantization box. Thus, an enhancement of the spontaneous emission rate is defined by the ratio electric field of the amplitudes of the functions ${\tilde{{\epsilon }}}^{(1)}$ and ${\tilde{{\epsilon }}}^{(2)}$ at the place of the emitter and their average values in the empty parts of the quantization box.

Figure 4a shows the profile of the field for the Tamm plasmon mode. It can be seen that the magnitude of the electric field at the layer adjacent to the silver layer (where the quantum dots are placed) increases substantially with respect to the field magnitude outside the structure. For the Tamm plasmon mode there is a fast exponential growth of the field near the metallic layer, characterized by the extinction coefficient of light in a metal. In the Bragg reflector, there is exponential growth of the magnitude of the photonic Bloch wave towards the quantum dots layers. Such increase in the magnitude of the field leading to a value of the modal Purcell factor as high as twenty, is responsible for the pronounced emission of light by the Tamm plasmon mode. It is interesting to note, that the growth of the magnitude of the Tamm plasmon mode towards the quantum dots is not monotonic: first, the field of the mode decays into the depth of the structure, reaches minimum, and then start to increase. This effect is explained by finite coherence length in the structure due to absorption: localized state, originated due to the interference, cannot occupy area larger than coherence length (in other words, monotonic exponential growth of the field profile envelope of eigen state, which in the photonic band gap is defined by the imaginary part of the Bloch wavevector, occurs only on the scale not exceeding the coherence length, limited by an absorption in the structure).

Figure 4b and c show the profiles of the field corresponding to the first and second edge state of the Bragg reflector^26, 27^. At the maxima, enhancements of the field are substantial, for the position of the quantum dots layers the magnitude of the field profile corresponds to those in uniform media. Finally, for the frequency, corresponding to the photonic band gap of the Bragg reflector (see Fig. 4d), amplitudes of the functions ${\tilde{{\epsilon }}}^{(1)}\,$and ${\tilde{{\epsilon }}}^{(2)}$ are reduced substantially.

## Conclusion

We have measured the photoluminescence pattern of the Tamm plasmon structures formed by GaAs/AlGaAs Bragg reflector containing InAs quantum dots as active area at room temperature. We have shown, that for Tamm plasmon modes photoluminescence intensity increases substantially. We have calculated spontaneous emission rate (modal Purcell factor) using S-quantization formalism, compared it with experimentally observed pholuminescence pattern, and by theoretical analysis of experimental results, that despite the absorption in the metallic layer, the spontaneous emission rate for the Tamm plasmon mode was increased by about one order of magnitude with respect to uniform media.

## Methods

### Theory

Probability of spontaneous emission has been calculated using S-quantization formalism^24^. Standard procedure of quantization of electromagnetic field imply setting of the periodic boundary conditions on the edges of the quantization box, which is equivalent to equating to unity the eigenvalues of the transfer matrix of the empty quantization box. An essence of S-quantization is the setting of eigenvalues of scattering matrix of system (inhomogeneous structure in the quantization box) to unity. S-quantization method allows to solve rigorously the problem of quantization of electromagnetic field in *non-uniform* media, avoid the problems of divergence of integral describing the effective mode volume and provide straightforward methods for calculating the probability of spontaneous emission for an arbitrary frequency of light, polarization and direction of propagation.

**Figure 5:**
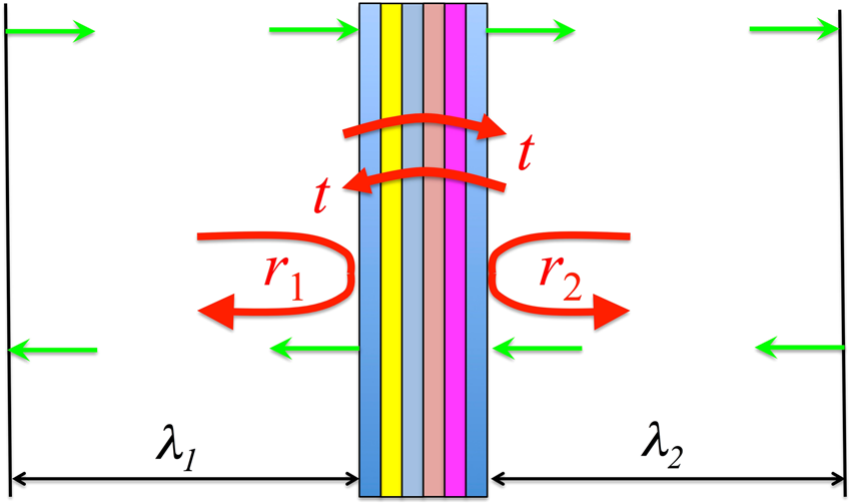
Illustration of S-quantization formalism: layered structure in the quantization box.

In the case of layered structure scattering matrix $\hat{S}$ has a dimensionality 2 × 2 and reads

3 $$\hat{S}=(\begin{array}{cc}\lambda t & {\lambda }_{2}^{2}{r}_{2}\\ {\lambda }_{1}^{2}{r}_{1} & \lambda t\end{array})$$

where *r*_1_and *r*_2_ are the amplitude reflection coefficients of the structure, *t* is the amplitude transmission coefficient, *λ*_1_ and *λ*_2_ are the phases gained by light propagating in left and right parts of the quantization box containing the structure (see Fig. 5), and *λ* = *λ*_1_ *λ*_2_.

Eigenvalues of $\hat{S}$ matrix reads

4 $${\beta }^{(1,2)}=\lambda (t\pm \sqrt{{r}_{1}{r}_{2}})$$

and related eigenvectors (describing the amplitude of the waves incident on the structure from the left and right sides of the quantization box) are

5 $${B}^{(1,2)}=[\begin{array}{cc}1, & \pm ({\lambda }_{1}/{\lambda }_{2})\sqrt{{r}_{1}/{r}_{2}\,}\end{array}]$$

Quantization of field implies equating of “incoming” and “outgoing” fields, which means equating the eigenvalues of the scattering matrix $\hat{S}$ to unity:

6 $${\beta }^{(1,2)}=1$$

When the size of the quantization box goes to infinity, mode spectrum becomes quasi-continuous, density of states become the same as for a uniform quantization box with periodic boundary conditions and insensitive to the quantization box size in specific structure of inhomogeneity, and probability of spontaneous emission is given by a spatial profile of the functions ${\tilde{\epsilon }}^{(1,2)}$ corresponding to the eigen-vectors (5).

It is convenient to introduce “modal Purcell factor” *F*. defined as a ratio of probability density of spontaneous emission for a specific frequency and direction of emission for the dipole, placed into the structure, to those in a uniform media *F*.

For TE polarized light, modal Purcell factor has a form

7 $${F}_{\theta }^{(TE)}=\frac{{|{\tilde{\epsilon }}^{(1)}|}^{2}+{|{\tilde{\epsilon }}^{(2)}|}^{2}}{{|{\epsilon }^{(1)}|}^{2}+{|{\epsilon }^{(2)}|}^{2}}$$

where function ${{\epsilon }}^{(1)}$ and ${{\epsilon }}^{(2)}$ describe electromagnetic field in uniform media. All functions ${{\epsilon }}^{(1,2)}$ are normalized using the same procedure: the modes corresponding to these functions are considered as elementary quantum oscillators with energy $\hslash \omega /2$ ^24^.

First an approach for the calculation of the Purcell factor based on the modification of electromagnetic mode spatial profile was introduced in^30^, but the periodic boundary conditions, which are inconsistent with the inhomogeneous structure in the quantization box were used in this paper. In brief, the formalism developed in^30^ can be obtained by using, instead two eigen vectors defined by (5), only one eigen vector *B* ^(1)^ with components [1,1]. At the end, an approach developed in ref. 30 works only for the structures possessing a centre of symmetry.

The transfer matrix method was used for the calculation of reflection, transmission and absorption spectra.

### Samples

**Figure 6:**
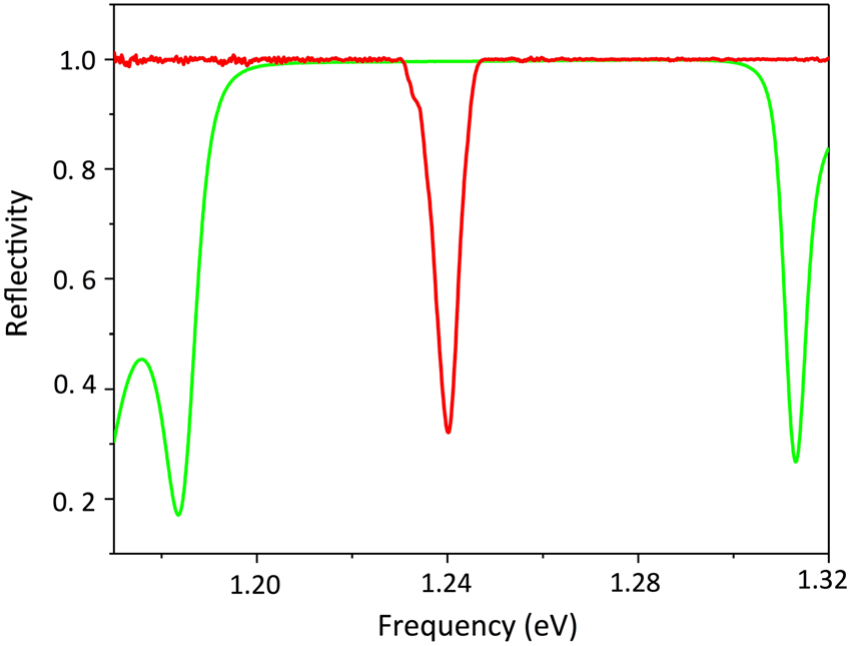
The reflection spectra of the Bragg reflector (green), before deposition of the silver layer, and the Tamm plasmon microcavity (red).

The sample under study is composed by a distributed Bragg reflector (DBR) formed by 30 pairs GaAs/Al_0.95_GaAs (with thicknesses 70 nm and 82 nm respectively) quarter-wavelength stacked layers grown on GaAs substrate by molecular beam epitaxy (MBE). In the upper high refractive index λ/4 layers three InAs monolayers (ML) were embedded to form quantum dots (QDs) as active medium, whose emission covers a wide wavelength range. The thicknesses of the layers in the Bragg reflector were chosen to provide a Bragg wavelength near 1000 nm. In order to form the Tamm plasmon (TP) state a 45 nm silver layer was deposited on top of the DBR. The thickness of the layer, adjacent to silver layer was reduced to 48 nm in line with^16, 31^ in order to match the spectral position of the Tamm plasmon mode to the centre of the photonic band gap, as shown in Fig. 6.

The layers of the DBR present a thickness gradient along the wafer, which enables a precise spectral tuning of the Tamm mode.

### Measurements

To perform angular resolved photoluminescence (PL) experiments, the sample was illuminated from the top by means of a Ti:Sapphire laser operating at 780 nm (pulse duration ∼200 fs, repetition rate 80 MHz), focused on the sample surface to a spot with a diameter ∼8 μm, via a long working distance microscope objective (NA = 0.75). An emitted light was collected using the same objective and sent to a spectrometer via an associated cooled charged coupled device (CCD). To image the angular dispersion of the sample reflectivity or emission the Fourier plane of the objective is imaged on the spectrometer entrance slit. The emission pattern presented in Fig. 2d was measured at room temperature, but for availability to perform at low temperature (77 K) the sample was mounted on the cold finger cryostat. Reflection spectra were measured using a similar scheme with the lamp as a source of light.

### Determination of the emission spectrum of bare quantum dots

**Figure 7:**
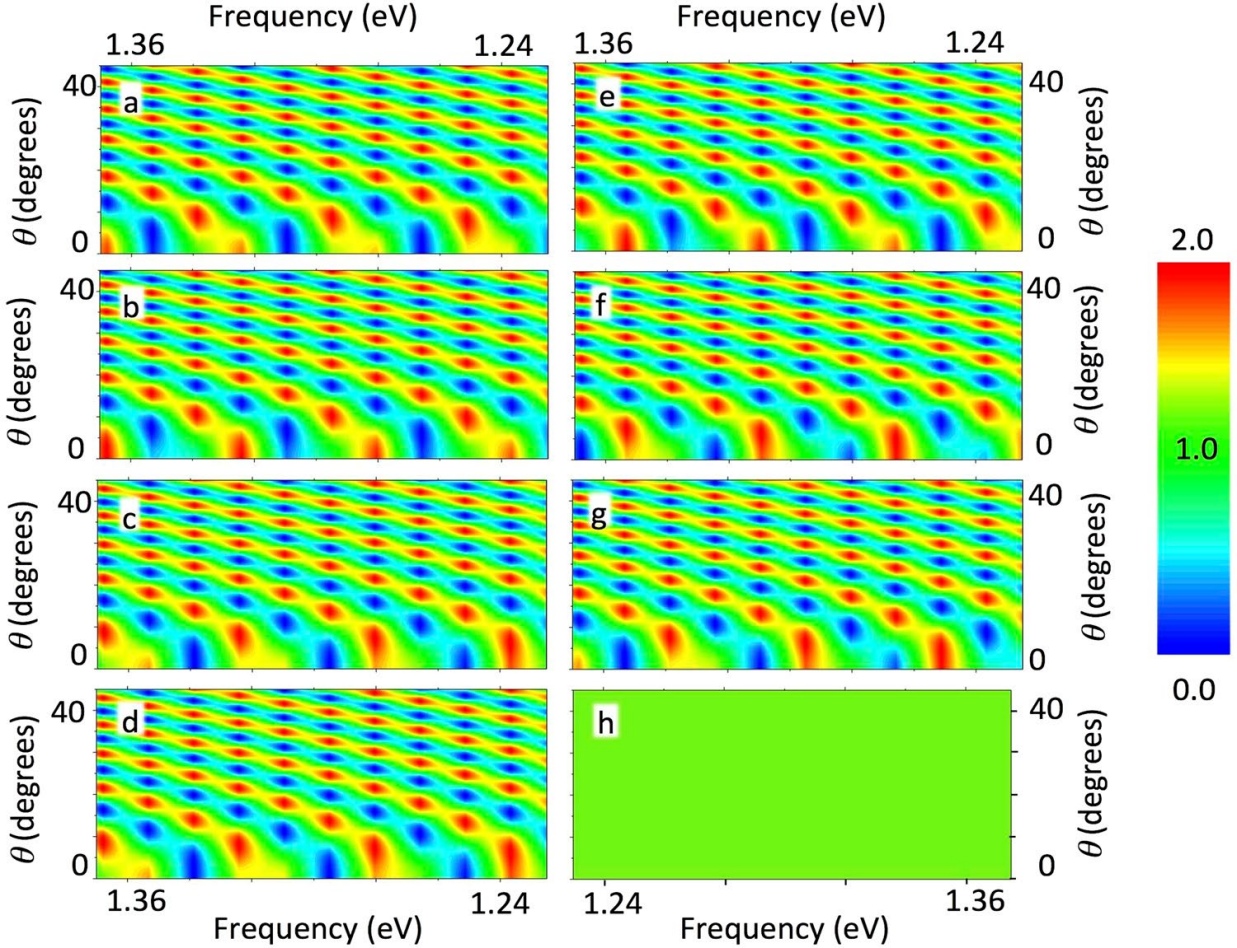
Dependence of the modal Purcell factor on frequency and direction of emission for a different position from the interface: (**a**) 17 nm; (**b**) 28 nm; (**c**) 35 nm; (**d**) 56 nm; (**e**) 71 nm; (**f**) 93 nm; (**g**) 142 nm; for the slab of GaAs of the thickness 28 μm surrounded by air. The Fig. 7 (**h**) demonstrates the pattern of modal Purcell factor averaged over the distance of the emitter from the interface on the interval corresponding to one wavelength.

In principle, any spatial inhomogeneity of a dielectric constant modifies the electromagnetic mode pattern, and consequently the spontaneous emission rate. In order to extract spectrum of quantum dots, not influenced by interfaces we have measured the emission spectrum from the edge of the structure. In this case, quantum dots positioned on a different depth from the interface contribute to the emission. The emission patterns for the emitters placed at different distances from the interface with air are shown in Fig. 7a–g together with the pattern averaged over a different position of emitter within one wavelength (Fig. 7h).

It can be seen, that the pattern for fixed positions of an emitter are nothing but a periodic function of the frequency of emission; the angular dependence also oscillates. The dependence of the frequency of such oscillation is governed by the factor cos(*kd*) where k is the wave vector of the light and *d* is the size of the sample.

Such patterns, averaged over all positions of emitter produce a uniform pattern. The calculations in the Fig. 7 were made for the structure of the thickness 28 μm for illustration purposes. For the realistic sample (with lateral size of few millimeters), the modal Purcell factor will oscillate very frequently with both, the size of the sample and the angle of emission, providing uniform pattern. This conclusion also holds for waveguided modes of the layered structure (including surface plasmon modes). Thus, the emission pattern from the edge of the sample, which is formed by quantum dots positioned at different distances from the interface, provided an experimentally measured emission spectrum, corresponds to the emission spectrum of bare quantum dots (which is similar to typical emission spectrum of bare quantum dots layer, demonstrated for example, in ref. 29).
